# Supplementary figures and images for: Allelic expression mapping across cellular lineages to establish impact of non-coding SNPs
Source: Mol Syst Biol. 2014 Oct 17;10(10):1–15. doi: 10.15252/msb.20145114 (PMC4299376; doi:10.15252/msb.20145114)

Figure S1. Rank order of associations in simulated candidate loci.

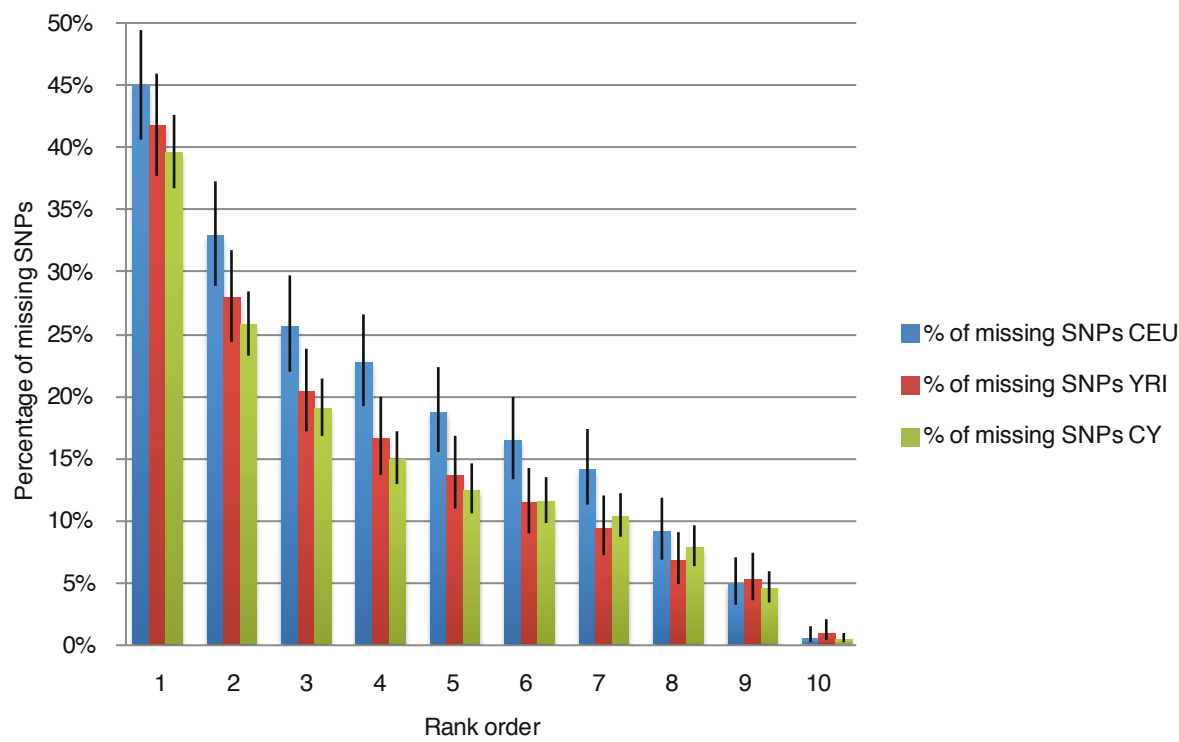

Supplement: Supplementary file 1 — Supplementary Figure S1 [file msb0010-0754-sd1.pdf]
