# Supplementary material for: Allelic expression mapping across cellular lineages to establish impact of non-coding SNPs
Source: Mol Syst Biol. 2014 Oct 17;10(10):1–15. doi: 10.15252/msb.20145114 (PMC4299376; doi:10.15252/msb.20145114)

Figure S2. Analysis of allelically regulated loci with shared cis-associations between populations revealed enriched networks.

Analysis generated through the use of IPA (Ingenuity® Systems, www.ingenuity.com).

CY= CEU LCLs and YRI LCLs shared associations.

CYM= CY and monocytes shared associations.

CYFM= shared associations between all cell-populations (CEU LCLs, YRI LCLs, fibroblasts and monocytes).

Score is based on algorithm that aggregates genes based on known relationships in the Ingenuity Knowledge Base.

Focus molecules = # of genes in dataset involved in this network

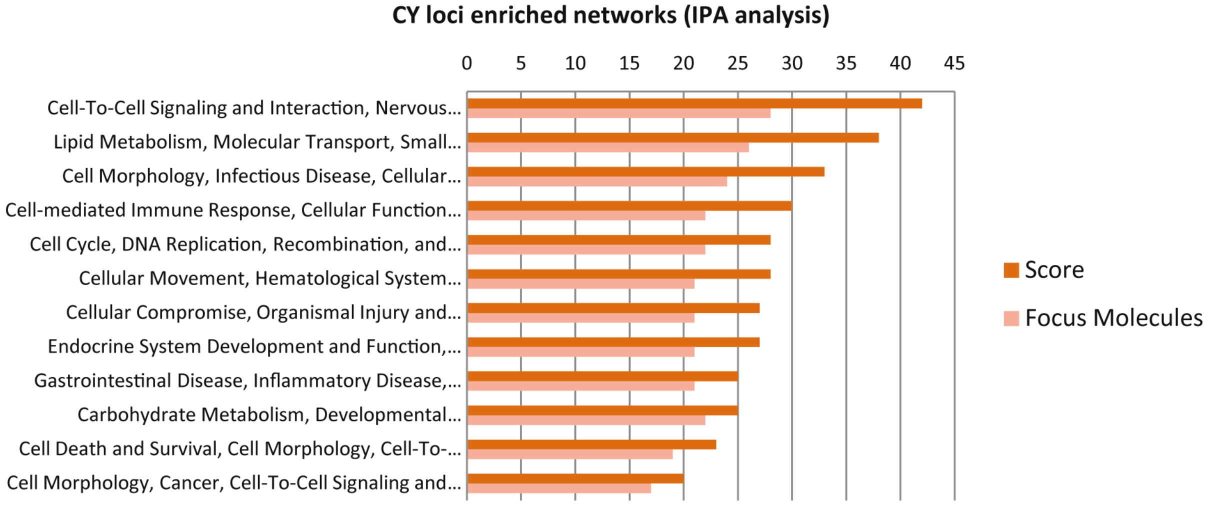

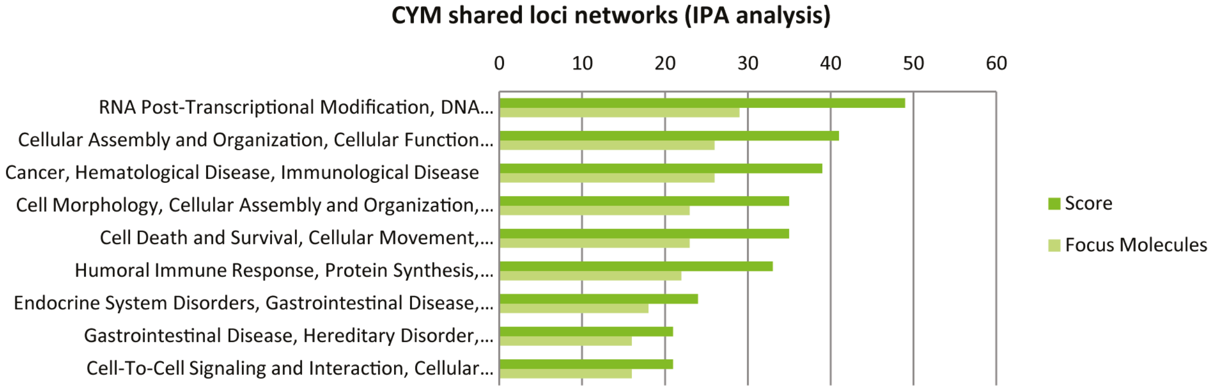

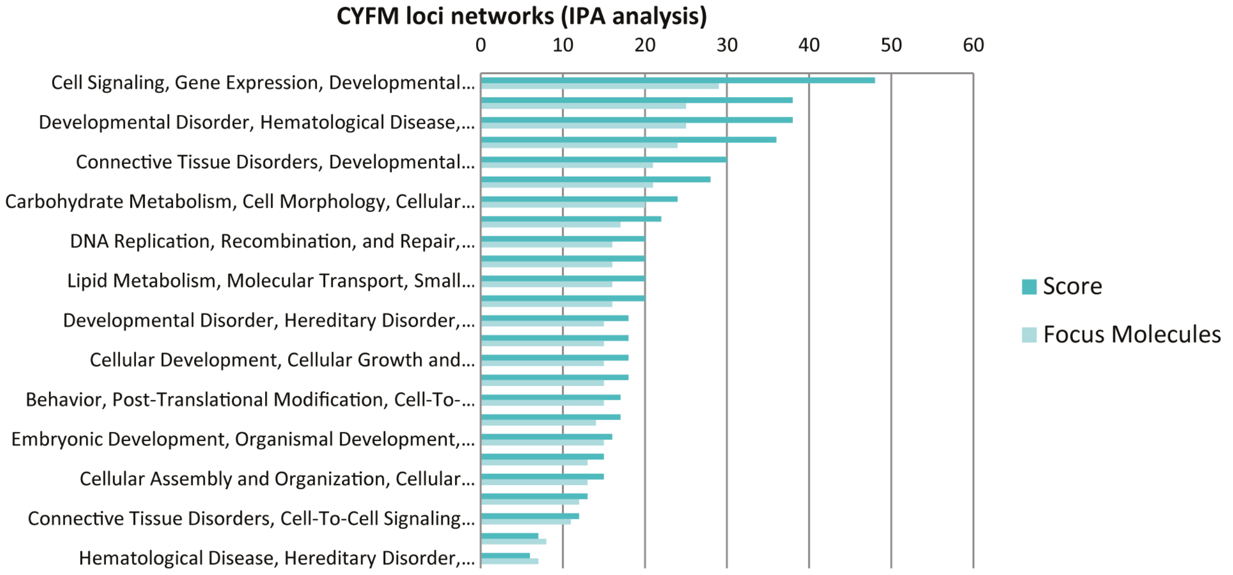

Supplement: Supplementary file 2 — Supplementary Figure S2 [file msb0010-0754-sd2.pdf]
