# Supplementary material for: Allelic expression mapping across cellular lineages to establish impact of non-coding SNPs
Source: Mol Syst Biol. 2014 Oct 17;10(10):1–15. doi: 10.15252/msb.20145114 (PMC4299376; doi:10.15252/msb.20145114)

Figure S3. Comparison of shared loci between full and equal size datasets.

A) Percentage of total shared associations between full size and equally powered datasets. We observe narrow range of variation of shared loci between the initial mapping and the bootstrapping analysis. For the selection of replicated and shared associations, see the Supplementary Methods.

B) Representation of differences in variance explained (r2) for monocyte associations shared or not shared with CEU LCLs, YRI LCLs and fibroblasts. We observe that shared associations with YRI LCLs show more divergence in variance explained. These data are representative also of others cell populations. This suggests that allelic expression architecture is further modulated by additional sequence variation in YRI at each locus.

A

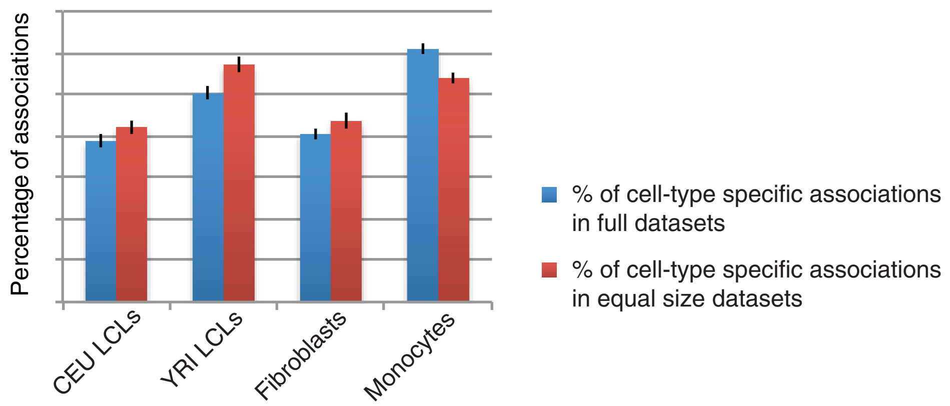

B

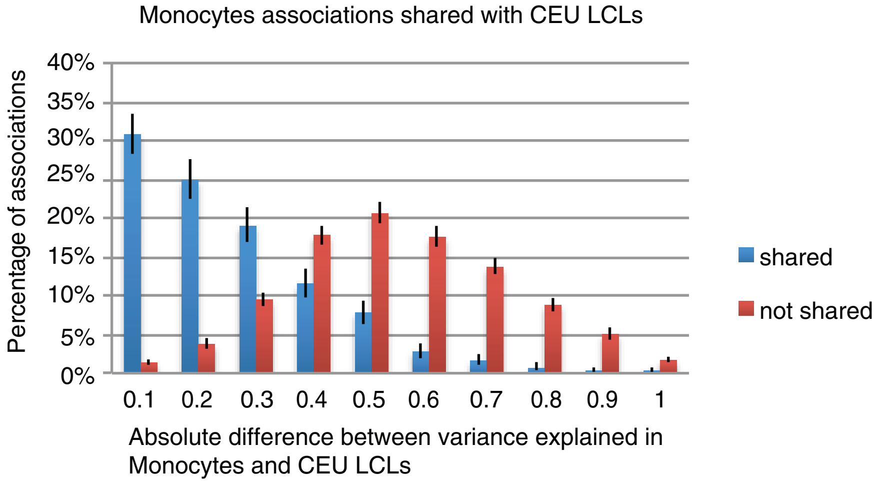

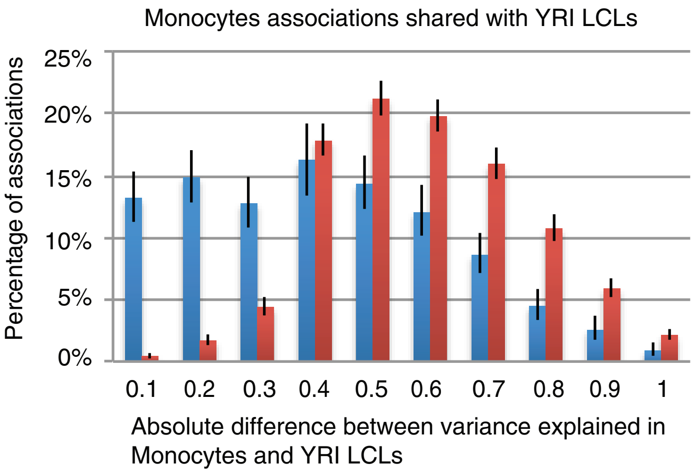

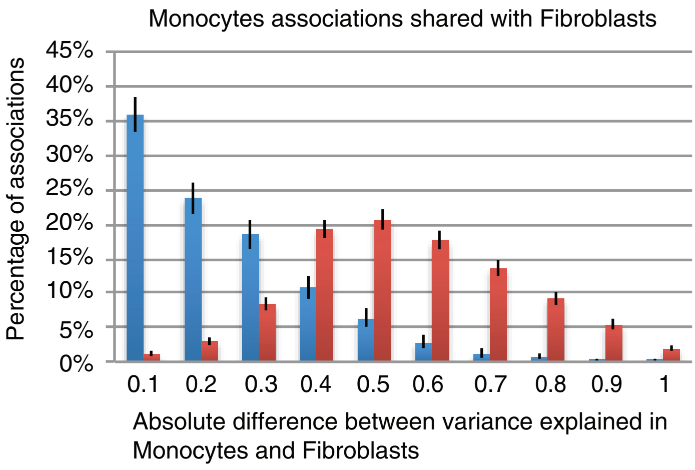

Supplement: Supplementary file 3 — Supplementary Figure S3 [file msb0010-0754-sd3.pdf]
