# Supplementary material for: Allelic expression mapping across cellular lineages to establish impact of non-coding SNPs
Source: Mol Syst Biol. 2014 Oct 17;10(10):1–15. doi: 10.15252/msb.20145114 (PMC4299376; doi:10.15252/msb.20145114)

Figure S4. Significant diminution of the total number of associated SNP per loci, example for loci with cis-rSNP shared between all populations ($P < 2.08 \times 10^{-32}$).

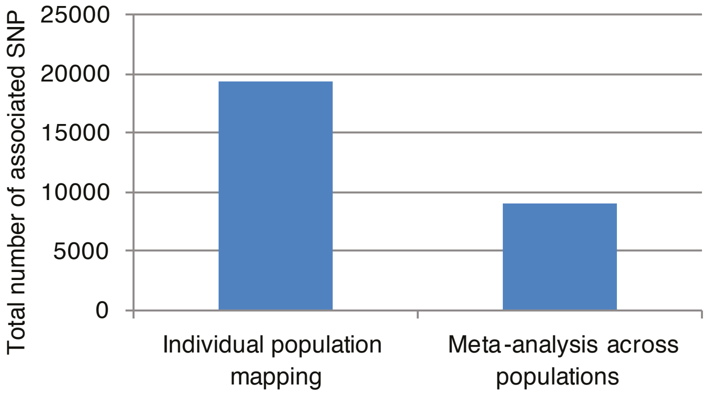

Supplement: Supplementary file 4 — Supplementary Figure S4 [file msb0010-0754-sd4.pdf]
