# Supplementary material for: Allelic expression mapping across cellular lineages to establish impact of non-coding SNPs
Source: Mol Syst Biol. 2014 Oct 17;10(10):1–15. doi: 10.15252/msb.20145114 (PMC4299376; doi:10.15252/msb.20145114)

Figure S5. Distribution of *cis*-rSNPs according to gene position.

Each transcription unit is divided into 3 parts according to critical regulatory regions (first exon, first intron, rest of the gene) in agreement with the literature (Veyrieras et al, 2012). Regions of 2 Kb upstream and downstream of the transcription unit are used to assess the promoter region and the region immediately downstream of the transcription end site (TES). Flanking regions of 10 Kb in size are assessed upstream of the TSS and downstream of the TES to a 100 kb distance from the transcript unit. Distribution of shared associations showed higher enrichment near the 5'UTR, where cell-specific associations are less frequent.

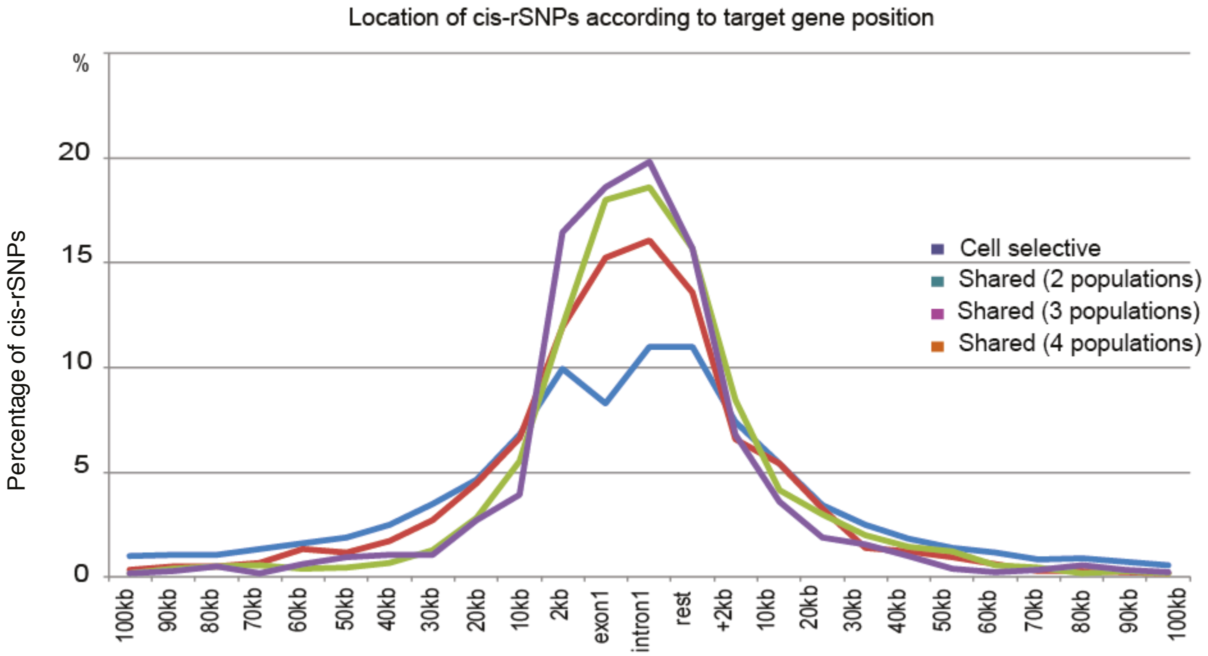

Supplement: Supplementary file 5 — Supplementary Figure S5 [file msb0010-0754-sd5.pdf]
