# Supplementary material for: Allelic expression mapping across cellular lineages to establish impact of non-coding SNPs
Source: Mol Syst Biol. 2014 Oct 17;10(10):1–15. doi: 10.15252/msb.20145114 (PMC4299376; doi:10.15252/msb.20145114)
Supplement: Supplementary file 6 — Supplementary Figure S6 [file msb0010-0754-sd6.pdf]

Figure S6. Examples of complex associations.

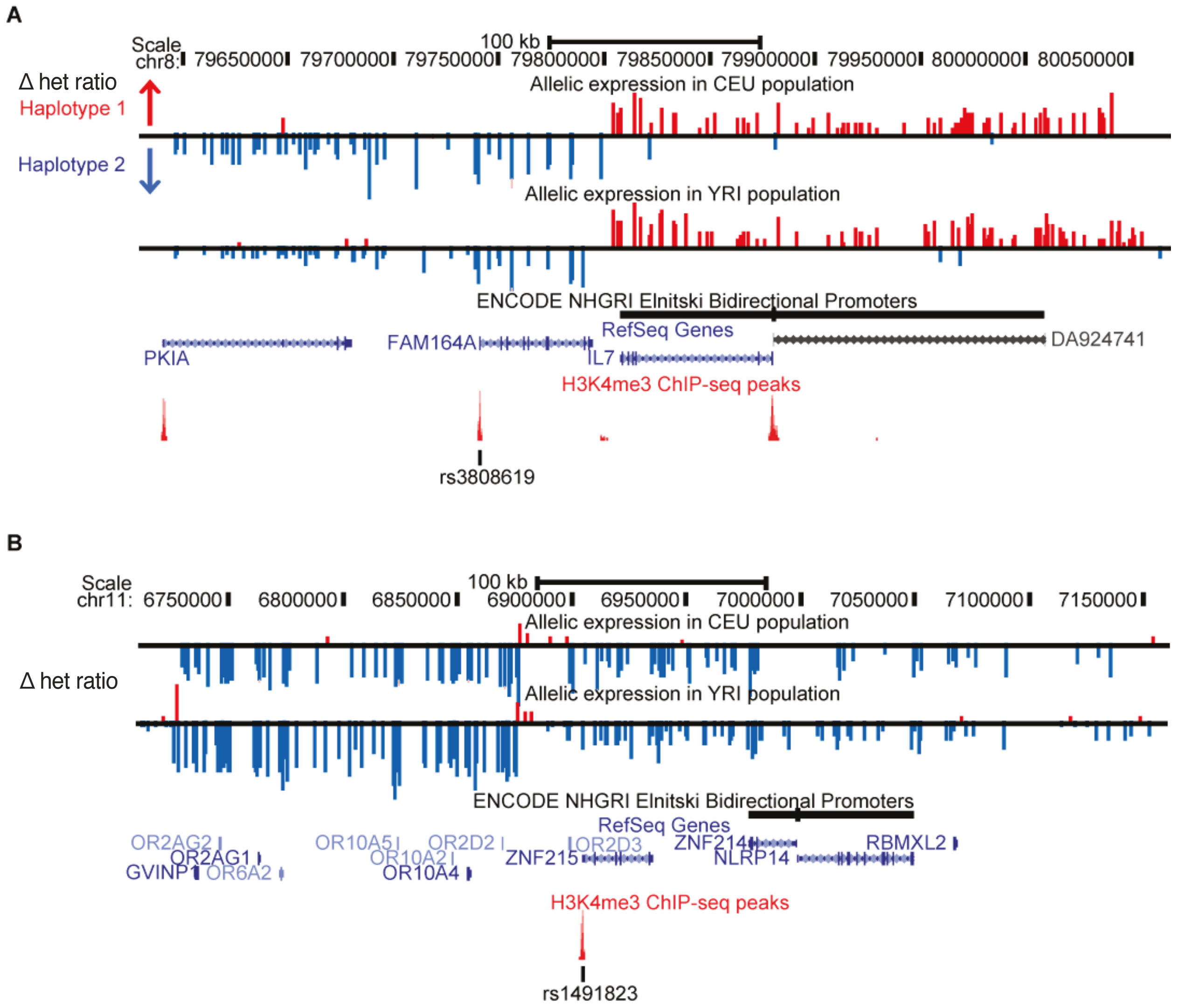

Here are depicted AE windows in the genomic region of two complex loci. On the top the mean Δhet ratios across measured SNPs in CEU and YRI populations (red=bias toward Haplotype 1; blue=bias toward haplotype 2), below the RefSeq genes and the bottom tracks show informative ChIPseq peaks as well as top *cis*-rSNP location.

(A) First locus located in chr8:79,579,086-80,068,368 is holding 3 Refseq genes and an unknown transcript sharing a bidirectional promoter with IL7 gene and all showed significant association to the same *cis*-rSNP (rs3808619 , P< 2.29 × E-09 for the RefSeq genes). This block of genes located in the middle of a gene's desert, the closer gene being found at more than 600kb, has been shown as linked with Multiple Sclerosis in GWAS study (Lin, Robben et al. 2008). We observed an opposite direction of effect between right and left sides of the locus pointing out a level of regulation even more complex.

(B) Second locus located in chr11:6,709,208-7,160,003 contains 12 referenced genes, with GVINP1, OR2D2, ZNF215, ZNF214, and NLRP14 (P< 5.6 × E-06) contained in the set of very high-confidence
