# Supplementary material for: Allelic expression mapping across cellular lineages to establish impact of non-coding SNPs
Source: Mol Syst Biol. 2014 Oct 17;10(10):1–15. doi: 10.15252/msb.20145114 (PMC4299376; doi:10.15252/msb.20145114)
Supplement: Supplementary file 7 — Supplementary Figure S7 [file msb0010-0754-sd7.pdf]

Figure S7. The master regulator rs3812560 is associated to differential AE of at least 5 genes and link to 4 auto-immune diseases.

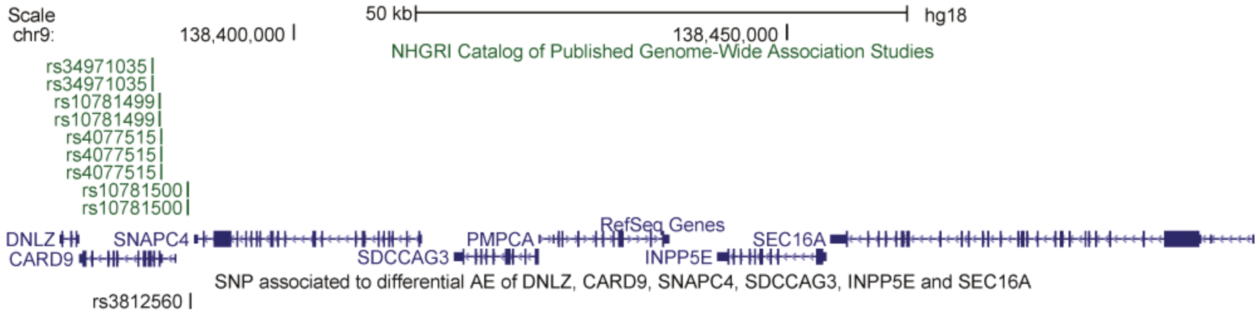

Gene	Acc	Orientation	Chr	Start	End	Population	Genotype associated to higher gene expression	P	Slope
DNLZ	ENST00000371738.3	-	9	1.39E+08	1.39E+08	M	C	8.76E-15	0.036
CARD9	ENST00000371734.3	-	9	1.39E+08	1.39E+08	M	C	2.14E-22	0.027
SNAPC4	ENST00000298532.2	-	9	1.39E+08	1.39E+08	CYFM	T	2.97E-19	-0.02
SDCCAG3	ENST00000371725.3	-	9	1.39E+08	1.39E+08	YF	T	7.52E-09	-0.021
INPP5E	ENST00000371712.3	-	9	1.39E+08	1.39E+08	FM	T	5.59E-33	-0.073

Variant rs3812560 (RegDG score=1f) is associated to differential allelic-expression of at least five genes: DNLZ, CARD9, SNAPC4, SDCCAG3 and INPP5E (last both not initially included in our analysis due to overlapping with other transcripts). It was mapped in different populations according to each transcript. The regulatory effect is opposite between the upstream genes (DNLZ and CARD9) and the downstream genes (SNAPC4, SDCCAG3 and INPP5E) relative to rs3812 560 location. This variant is in strong LD with: rs10781499 (RegDB=NA) which is linked to ulcerative colitis risk and inflammatory bowel disease; rs10781500 (RegDB=6) a variant associated to ankylosing spondylitis and ulcerative colitis; and rs4077515 (Reg DB=1f) a SNP linked to Crohn's disease susceptibility. Mainly because of its position and function, CARD9 was thought to be the candidate gene for these auto-immune diseases (Pointon et al. 2010). However, since SDCCAG3 may be involved in the modulation of TNF response, a strong immune component, it could also possibly be associated to these diseases. Supplementary work would be required to validate this hypothesis.
