# Supplementary material for: Allelic expression mapping across cellular lineages to establish impact of non-coding SNPs
Source: Mol Syst Biol. 2014 Oct 17;10(10):1–15. doi: 10.15252/msb.20145114 (PMC4299376; doi:10.15252/msb.20145114)

Figure S8. Validation of the inhibition of NFkB

Significant changes upon perturbation of NFkB on known gene targets include Il-6 (P=0.00454), Il-8 (P=0.0097), Il-1a (P=0.0308), and Bcl-2 (P=0.0021). P-values were calculated using Fisher's exact test across five samples: GM12878, GM12891, GM12892, GM19240, GM19239, and GM19238.

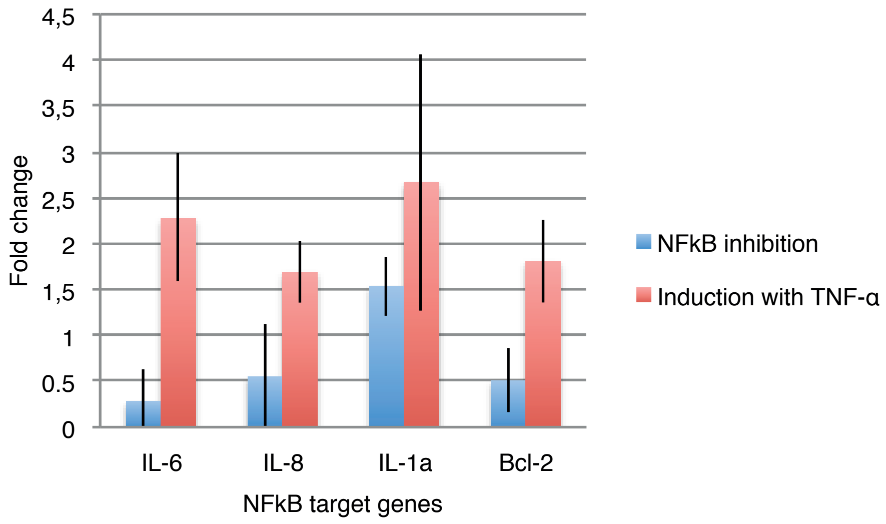

Supplement: Supplementary file 8 — Supplementary Figure S8 [file msb0010-0754-sd8.pdf]
