# Supplementary material for: Allelic expression mapping across cellular lineages to establish impact of non-coding SNPs
Source: Mol Syst Biol. 2014 Oct 17;10(10):1–15. doi: 10.15252/msb.20145114 (PMC4299376; doi:10.15252/msb.20145114)
Supplement: Supplementary file 9 — Supplementary Figure S9 [file msb0010-0754-sd9.pdf]

**Figure S9. Example of disease associated SNP linked to differential AE of target gene through NFkB action.**

(A) AE linear regression graph. (B) SERPIN1 locus.

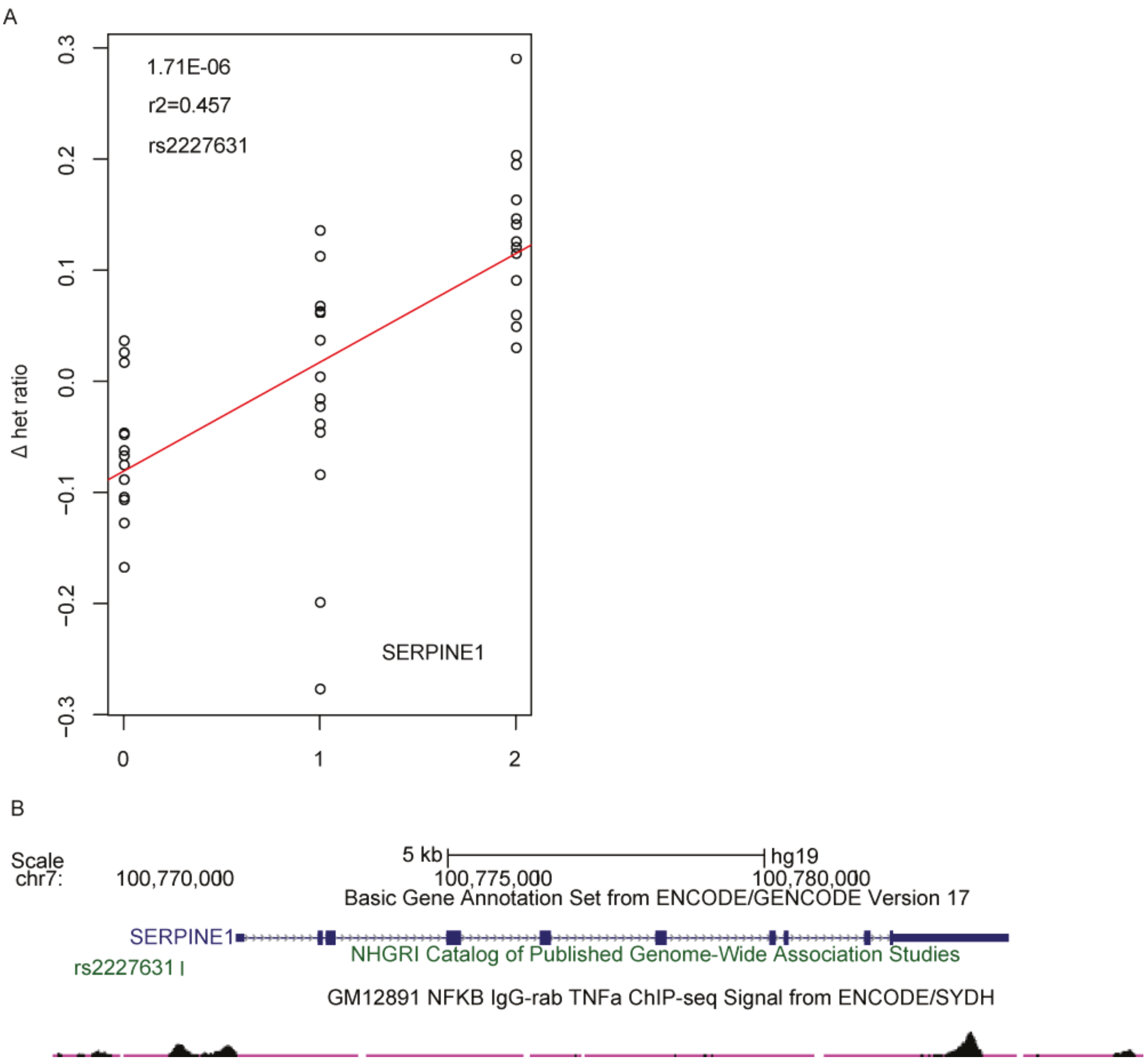


Mapped rs2227631 show strong association with differential AE of SERPINE1 (P= 1.71 × E-06 in CEU population). It has been shown as linked to circulating plasminogen activator inhibitor-1 (PAI-1) concentration and sit on an NFkB ChIP-seq peak.
