# Supplementary material for: Allelic expression mapping across cellular lineages to establish impact of non-coding SNPs
Source: Mol Syst Biol. 2014 Oct 17;10(10):1–15. doi: 10.15252/msb.20145114 (PMC4299376; doi:10.15252/msb.20145114)
Supplement: Supplementary file 20 — Supplementary Methods [file msb0010-0754-sd20.docx]

# Supplementary Methods.

Simulation to evaluate mapping accuracy

The allelic expression phenotypes we mapped have very high effect size and were detected in modest sample. This may raise concern of the impact of small fluctuations in linkage disequilibrium to accuracy of mapping. Furthermore, the mapping was based on imputated genotypes using 1000 Genomes with introducing small number of genotyping errors. To assess these effects to understand how causal SNPs would behave in our test we carried out 100 sets of simulated associations across 3000 randomly selected loci separately in CEU and YRI haplotype structures. The estimation in imputation was based on ~600K SNPs genotyped directly on Illumina Human 2.5M arrays (data provided by Dr. Eberle at Illumina Inc.) in all YRI and CEU trios but not included in imputation input data from HapMap Phase II data. The simulations took advantage of allelic expression phenotype distributions observed in mapped loci. The original cis-rSNP associations were binned based on MAF to 40 bins (0.1 – 0.5, with 0.025 bin size) and then a random locus in genome with directly genotyped site in appropriate MAF bin was assigned as causal SNP. The allelic expression distribution observed at mapped site heterozygotes and homozygotes were assigned to sample population based on random SNP genotype data and mapping of the effect in similarly sized window as in actual data was performed. From 30,000 such simulations in both populations we then assessed the rank order of each simulated causal SNP in the mapping datasets. We further combined mapping data in subset of loci where the simulated SNP was assigned to be same in CEU and YRI (shared). The results in supplementary figure 1 show that if we focused on only on SNPs ranked on top we would miss 38 – 42% of true causal SNP, but if we include top 10 SNPs for consideration based on mapping ranking the likelihood of missing the causal site is low (close to 1%). In addition, we observe that mapping accuracy is better in YRI, which is likely due to greater recombination (shorter LD blocks) as well as slightly larger sample size. Finally, at sites that are shared between 2 populations the combined mapping accuracy outperforms individual population mapping results as expected.

Bootstrapping analysis of mapped associations

To estimate variation in sharing between tissues in equally powered datasets, we re-ran top SNP-transcript associations using 10 sets of 45 samples randomly selected from each cell population. We used the same transcript list that was used for the full datasets, and all SNPs with maf >= 0.05 and located +/- 250kb from a gene. To select for replicated associations, we used p-value of most significant SNP for each loci and in each sets. For replication in primary cell population we used following criteria: at least one of ten p-values was < 1% FDR and median of 10 p-values was lower than 10% FDR. We then observed with replicated associations in bootstrapped data how they were overlapping other tissues/populations in original data. The replicated associations are showing similar levels of sharing as original associations in full samples indicating that larger size of sample did not lead to discovery of disproportionate number of tissue-specific associations or vice-a-versa (Figure S2A). Therefore, the variation observed between cell-types appears true and not driven by differential power. The bootstrapping analysis also allowed us to compare sharing between populations (Caucasian versus YRI) versus sharing between tissues. We observed that for shared associations, the YRI LCLs show more divergence in variance explained, suggesting that allelic expression architecture is further modulated by additional sequence variation in YRI at each loci (Figure S2B).
